# SecA Cotranslationally Interacts with Nascent Substrate Proteins *In Vivo*

**DOI:** 10.1128/JB.00622-16

**Published:** 2016-12-28

**Authors:** Damon Huber, Mohammed Jamshad, Ruby Hanmer, Daniela Schibich, Kristina Döring, Isabella Marcomini, Günter Kramer, Bernd Bukau

**Affiliations:** a Institute for Microbiology and Infection, School of Biosciences, University of Birmingham, Birmingham, United Kingdom; b Center for Molecular Biology of Heidelberg University (ZMBH), DKFZ-ZMBH Alliance, Heidelberg, Germany; c German Cancer Research Center (DKFZ), Heidelberg, Germany; Princeton University

**Keywords:** Sec, SecA, cotranslational translocation, posttranslational translocation, protein targeting, protein translocation, secretory pathway

## Abstract

SecA is an essential component of the Sec machinery in bacteria, which is responsible for transporting proteins across the cytoplasmic membrane. Recent work from our laboratory indicates that SecA binds to ribosomes. Here, we used two different approaches to demonstrate that SecA also interacts with nascent polypeptides *in vivo* and that these polypeptides are Sec substrates. First, we photo-cross-linked SecA to ribosomes *in vivo* and identified mRNAs that copurify with SecA. Microarray analysis of the copurifying mRNAs indicated a strong enrichment for proteins containing Sec-targeting sequences. Second, we used a 2-dimensional (2-D) gel approach to analyze radioactively labeled nascent polypeptides that copurify with SecA, including maltose binding protein, a well-characterized SecA substrate. The interaction of SecA with nascent chains was not strongly affected in cells lacking SecB or trigger factor, both of which also interact with nascent Sec substrates. Indeed, the ability of SecB to interact with nascent chains was disrupted in strains in which the interaction between SecA and the ribosome was defective. Analysis of the interaction of SecA with purified ribosomes containing arrested nascent chains *in vitro* indicates that SecA can begin to interact with a variety of nascent chains when they reach a length of ∼110 amino acids, which is considerably shorter than the length required for interaction with SecB. Our results suggest that SecA cotranslationally recognizes nascent Sec substrates and that this recognition could be required for the efficient delivery of these proteins to the membrane-embedded Sec machinery.

**IMPORTANCE** SecA is an ATPase that provides the energy for the translocation of proteins across the cytoplasmic membrane by the Sec machinery in bacteria. The translocation of most of these proteins is uncoupled from protein synthesis and is frequently described as “posttranslational.” Here, we show that SecA interacts with nascent Sec substrates. This interaction is not dependent on SecB or trigger factor, which also interact with nascent Sec substrates. Moreover, the interaction of SecB with nascent polypeptides is dependent on the interaction of SecA with the ribosome, suggesting that interaction of the nascent chain with SecA precedes interaction with SecB. Our results suggest that SecA could recognize substrate proteins cotranslationally in order to efficiently target them for uncoupled protein translocation.

## INTRODUCTION

The Sec machinery is responsible for transporting proteins across and inserting proteins into the cytoplasmic membrane (1, 2). At its core, this machinery consists of an evolutionarily conserved protein complex (SecYEG in bacteria; Sec61p in eukaryotes) that forms a protein-conducting channel in the cytoplasmic membrane (3). Protein substrates of the Sec machinery pass through the SecYEG channel in an unfolded conformation during translocation across the membrane. However, proteins that fold before they can be translocated through SecYEG become trapped in the cytoplasm (4).

In bacteria, there are two distinct modes of translocation through SecYEG: (i) translationally coupled and (ii) translationally uncoupled (5). In the first mode, substrate proteins are recognized at an early stage in translation by the signal recognition particle (SRP), and the translating ribosome is coupled to SecYEG. Translocation through SecYEG is thought to be driven by binding of the ribosome directly to SecYEG (5, 6). Due to the tight coupling between synthesis and translocation, this mode is often referred to as “cotranslational translocation.” In order to distinguish it from this coupled pathway, the second mode is frequently referred to as the “posttranslational translocation.” Indeed, because translocation is not directly coupled to synthesis, most substrates of this mode engage SecYEG after the completion of protein synthesis (7). Nonetheless, many can engage SecYEG cotranslationally, albeit relatively late in the process of protein synthesis (7). Thus, in order to avoid confusion, we refer to this mode of translocation as “uncoupled” translocation and to SRP-dependent cotranslational translocation as “coupled” translocation.

In addition to SecYEG, most Sec substrates also require the assistance of the ATPase SecA, a motor protein that binds to SecYEG and drives protein translocation. All proteins that are exported by the uncoupled pathway require the assistance of SecA (1, 8, 9). SecA also appears to play a role in the translocation of at least a subset of substrates of the coupled translocation pathway (10–14). However, the role of SecA in coupled translocation is unclear. For example, biophysical experiments suggest that the binding of SecA and of ribosomes to SecYEG is mutually exclusive (15).

The coupled pathway appears to be primarily responsible for the translocation of integral cytoplasmic membrane proteins (IMPs), while the uncoupled pathway is mainly responsible for the export of outer membrane proteins (OMPs), soluble periplasmic proteins, and lipoproteins (16, 17). However, there is significant overlap between these two subsets. In both cases, substrate proteins are recognized by an internally encoded peptide signal (18). In IMPs, the signal is encoded within one of its transmembrane domains (TMDs) (19, 20). In the case of OMPs, soluble periplasmic proteins, and lipoproteins, this signal is encoded in an N-terminal signal sequence (SS), which is cleaved from the protein during translocation (21).

At least two other proteins, SecB and trigger factor (TF), interact with nascent substrates of the uncoupled pathway and could be involved in targeting substrate proteins to the uncoupled pathway (22, 23). SecB is a molecular chaperone that interacts with a subset of nascent and full-length substrates of the uncoupled Sec pathway and holds them in an unfolded conformation in the cytoplasm (4, 23–25). The interaction of SecB with SecA has led to the suggestion that SecB recognizes Sec substrates and delivers them to SecA (26, 27). TF is a ribosome-associated chaperone that interacts with a broad range of nascent substrate proteins, but it appears to interact preferentially with nascent OMPs *in vivo* (22). Mutations in the gene encoding TF (*tig*) improve the efficiency of translocation (22, 28) and can suppress the translocation defect of a *secB* mutant (29, 30), suggesting that TF antagonizes uncoupled translocation.

We recently published evidence that SecA binds to ribosomes very near to the TF-binding site (31, 32) and that this interaction is required for efficient translocation, raising the possibility that SecA interacts with nascent Sec substrates *in vivo*. In this work, we have investigated the interaction of SecA with nascent substrate proteins *in vivo* using two different approaches. In addition, we have examined the interaction of SecA with purified ribosomes containing arrested nascent chains of various lengths and the effects of SecB and TF on these interactions. Our results indicate that SecA binds specifically to a broad range of nascent Sec substrates and that it can begin to interact with nascent substrate proteins when they reach a length of ∼100 amino acids. Finally, our results suggest that the interplay between SecA, SecB, and TF, which is important for determining the timing of protein translocation, is complex.

## RESULTS

### Site-specific cross-linking of SecA to nascent substrate proteins *in vivo*.

We reasoned that if SecA interacts with nascent substrate proteins, it should copurify with mRNAs encoding these proteins. Because the affinity of SecA for substrate proteins is relatively low (43), we stabilized the interaction between SecA and substrate proteins by introducing a UV-activatable cross-linking agent, benzophenylalanine (Bpa), *in vivo* at position 796 of SecA using nonsense suppression (33). Position 796 (glutamine) is located in the 2-helix finger of SecA (34), which contacts substrate proteins during protein translocation, and it is directly adjacent to alanine-795, which was shown previously to contact substrate proteins using disulfide cross-linking (35). In addition, we used a His_6_-affinity-tagged SUMO-SecA fusion protein (His-SUMO-SecA796*) in order to facilitate purification of cross-linking products.

**FIG 1:**
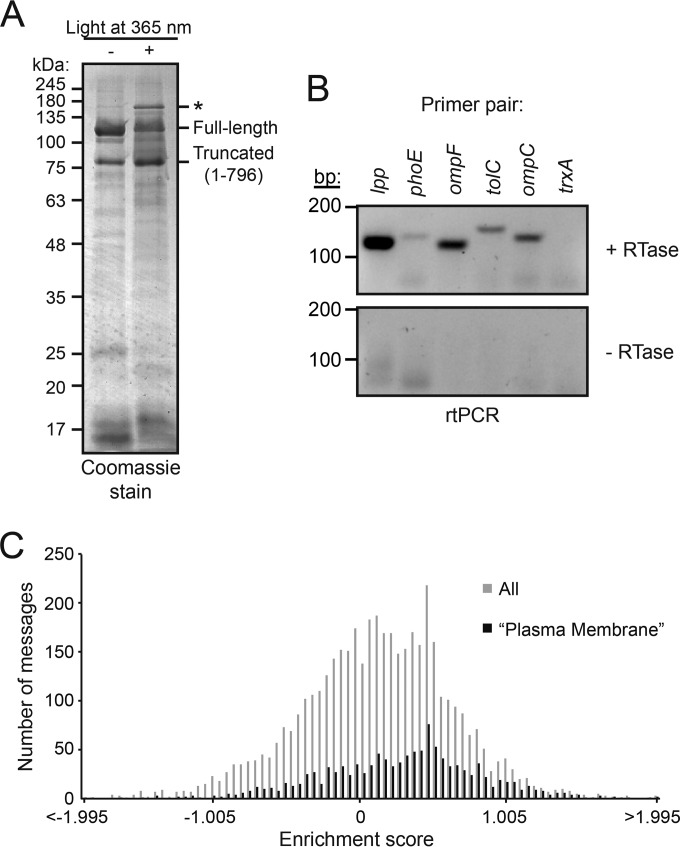
SecA copurifies with mRNAs encoding nascent Sec substrates. (A) Cells expressing His-SUMO-SecA796* and an orthologous tRNA-tRNA synthetase system evolved to recognize Bpa were incubated in the presence (+) or absence (−) of UV light at 365 nm. Treatment with UV light resulted in the appearance of at least one high-molecular-mass adduct (*), which was purified using an Ni affinity column. The running positions of full-length His-SUMO-SecA (Full-length) and the truncated peptide in which translation terminated at codon 796 (Truncated) are indicated. (B) RT-PCR analysis of UV-treated, StrepTactin-purified Strep-SUMO-SecA796* using primers specific for the messages encoding Braun's lipoprotein (*lpp*), PhoE, OmpF, TolC, OmpC, and thioredoxin-1 (*trxA*). Top, reaction mixtures containing complete RT-PCR mixture (including reverse transcriptase). Bottom, PCR mixtures in which reverse transcriptase was omitted. (C) Histogram of enrichment scores for mRNAs that copurify with SecA. Strep-SUMO-SecA796* was cross-linked to ribosomes as in the experiment whose results are shown in panel A, ribosomes were purified from cell lysates by ultracentrifugation (total ribosomes), and the Strep-SUMO-SecA796*-cross-linked ribosomes were purified using a StrepTactin column (SecA-cross-linked ribosomes). The mRNAs from the total ribosomes and SecA-cross-linked ribosomes were isolated by RT-PCR using a poly(A) primer and hybridized to an E. coli microarray. The enrichment score is the log_2_ of the signal from the purified SecA-cross-linked fraction divided by the signal from total ribosome samples. Gray bars, all messages; black bars, messages for proteins in gene ontology category GO:0005886 (plasma membrane).

Exposing cells expressing His-SUMO-SecA796* to light at 365 nm resulted in the appearance of a prominent high-molecular-mass adduct, which was purified using Ni^2+^ affinity chromatography (Fig. 1A). The adduct cross-reacted with an anti-SecA antibody, indicating that it contained SecA (see Fig. S1A in the supplemental material). N-end sequencing identified a peptide with the sequence AEIYNKD, consistent with the presence of mature-length OmpF, and analysis of the most prominent adduct by mass spectrometry indicated the presence of OmpF (see Fig. S1B).

In order to investigate whether SecA interacts with nascent polypeptides, we analyzed the mRNA content of the purified cross-linking adducts. In order to prevent the spurious interaction of ribosomes with the affinity resin, we used a variant of SecA796* containing a triple Strep-tag II (Strep-SUMO-SecA796*) instead of His-SUMO-SecA796* (see Fig. S1C in the supplemental material). We then carried out reverse transcription PCR (RT-PCR) on the purified sample using primers specific for the mRNAs encoding OmpF, OmpC, PhoE, TolC, and Lpp (Fig. 1B, top, first 5 lanes). All five primer pairs produced PCR products, and the level of the products approximately reflected the relative expression levels of each gene. The omission of reverse transcriptase from the PCR mixture eliminated the presence of a PCR product (Fig. 1B, bottom), indicating that the products were not the result of contaminating chromosomal DNA. Primers specific for the mRNA encoding a prominent cytoplasmic protein, thioredoxin-1, did not yield a PCR product (Fig. 1B, 6th lane).

### Identification of mRNAs that copurify with nascent-chain-cross-linked SecA.

In order to identify the transcripts to which SecA cross-linked, we purified Strep-SUMO-SecA796*-cross-linked ribosomes. To this end, we isolated ribosomes from photo-cross-linked cell lysates by centrifugation through a sucrose cushion (total ribosome sample). We then purified the SecA-cross-linked ribosomes using a StrepTactin affinity column. The addition of buffer containing desthiobiotin resulted in elution of ribosomes in the expected fractions (see Fig. S1D in the supplemental material), and the presence of SecA-cross-linking adducts was confirmed by Western blotting (see Fig. S1E). No proteins were eluted when the same procedure was carried out using lysates from cells producing His-SUMO-SecA796* as a negative control. We analyzed the cDNA produced from the total ribosome fraction and the purified SecA-cross-linked ribosome fraction using an Escherichia coli microarray. A histogram of the enrichment scores suggests a bimodal or heavily right-skewed distribution (Fig. 1C, gray bars). Several lines of evidence suggest that this skew is due to the enrichment of messages encoding IMPs. First, of the 124 most enriched messages, 79 encoded integral membrane proteins and 9 encoded other cell envelope proteins containing N-terminal signal sequences (soluble periplasmic proteins, lipoproteins, and outer membrane proteins). Of the remaining messages, 21 are located in the same polycistronic message as an integral membrane or cell envelope protein. Second, the average enrichment score for messages encoding proteins with the gene ontology (GO) term GO:0005886 (Plasma Membrane) was significantly higher than that of the remaining cytoplasmic proteins (0.27 compared to 0.06; *P* = 2 × 10^−29^). The enrichment of this subpopulation is clear from the histogram of their scores (Fig. 1C, black bars). Indeed, of the 50 least enriched messages encoding proteins with the term GO:0005886, 24 actually encoded soluble proteins (e.g., the ATP-binding proteins or periplasmic binding proteins of ABC transporters), suggesting that this enrichment is even stronger than our analysis indicates. Finally, a comparison of (i) the number of transmembrane domains, (ii) the size of the largest soluble periplasmic domain, and (iii) the topology of the N terminus suggests that the only feature that was significantly different between the 50 most and the 50 least enriched IMPs was the number of transmembrane domains: the most enriched IMPs contained an average of 9.6 (median of 11) transmembrane domains, and the least enriched contained an average of 6.1 (median of 5.5) transmembrane domains (*P* = 6.5 × 10^−6^). The average score for messages encoding proteins with term GO:0042597 (Periplasmic Space) (0.01), which includes OMPs, soluble periplasmic proteins, and lipoproteins, was not significantly different from that of cytoplasmic proteins.

### 2-D gel analysis of the interaction of SecA with nascent substrate proteins.

**FIG 2:**
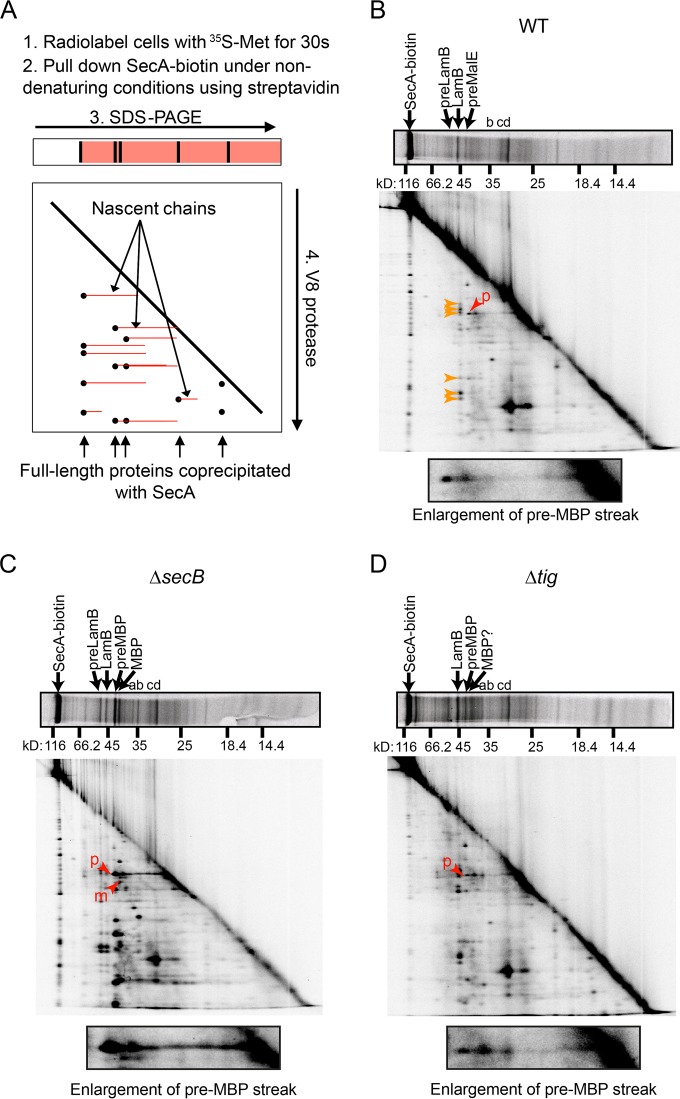
2-D gel analysis of the interaction of SecA-biotin with nascent polypeptide *in vivo*. (A) Diagrammatic representation of 2-D gel analysis. Cells expressing SecA-biotin as their sole copy of SecA were grown in M63 maltose containing IPTG and radiolabeled for 30 s with ^35^S-methionine, and SecA-biotin was pulled down from the cell lysates under nondenaturing conditions. The proteins that coprecipitated with SecA-biotin were resolved in the first dimension using SDS-PAGE. Gel slices containing the resolved proteins were then loaded on to a second gel and subjected to in-gel proteolysis using the V8 protease. Proteolytic fragments of full-length proteins (black) resolve as spots running below the full-length bands, while nascent polypeptides (red) containing the same proteolytic fragments resolve as streaks extending back from these spots in the second dimension. (B to D) 2-D gel analysis was carried out on cells expressing SecA-biotin as the sole copy of SecA (DRH839) (B) and on Δ*secB* (DRH841) (C) and Δ*tig* (DRH866) (D) mutants of DRH839. The running positions of full-length precursor LamB (preLamB), mature LamB, pre-MBP, and mature MBP are indicated in the first dimension. Full-length bands with molecular masses corresponding to precursor OmpC (a), mature OmpC (b), precursor OmpA (c), and mature OmpA (d) are indicated. Red arrowheads in the second dimension indicate the N-terminal peptide fragments of preMBP (p) and mature MBP (m). Orange arrowheads indicate the peptide fragments generated by full-length LamB. Question marks indicate that identification of the full-length species or peptide fragment could not be made unambiguously due to lack of signal. An enlargement of the region of the gel corresponding to the N-terminal proteolytic fragment of precursor-length MBP is depicted below each 2-D gel.

In a complementary line of experimentation, we examined the ability of SecA to interact with nascent polypeptides using a 2-dimensional (2-D) gel method originally devised by Kumamoto and Francetic (23) and Chun and Randall (36) (depicted diagrammatically in Fig. 2A). We labeled newly synthesized proteins using [^35^S]methionine and purified SecA from the cell lysates. To this end, we used cells in which the sole copy of the *secA* gene produces a variant of SecA that is covalently linked to biotin at its C terminus (SecA-biotin) (32, 37). Purification using a C-terminal tag avoided purification of nascent SecA species, which would complicate the interpretation of our results. SecA-biotin was functional as indicated by the normal temporal processing of the maltose binding protein (MBP) in these strains (compare the processing of MBP in Fig. S2 in the supplemental material to that in Josefsson and Randall [38]). After streptavidin affinity purification, we separated SecA and the copurifying polypeptides according to size in the first dimension using SDS-PAGE. Nascent species of a single protein consist of a range of polypeptides with different molecular masses and are difficult to resolve by standard SDS-PAGE. In order to resolve the nascent species, we subjected the entire lane to in-gel proteolysis using a site-specific protease and separated the proteolytic fragments according to size in a second SDS-PAGE dimension.

Among the copurifying proteins, we identified MBP and LamB based on the molecular masses of the full-length species in the first dimension (Fig. 2B, top) and the proteolytic digestion pattern in the second dimension (compare the digestion patterns in Fig. 2 to those of immunoprecipitated LamB and MBP in Fig. S2 in the supplemental material). In the case of MBP, SecA-biotin appears to interact predominantly with the unprocessed, precursor-length species. In addition, we could tentatively identify two other full-length proteins as OmpC and OmpA by comparing their digestion patterns to those published previously (23). For many of the copurifying proteins, there were horizontal streaks extending toward lower molecular masses in the first dimension (toward the right in Fig. 2B), suggesting the presence of nascent species of these proteins. The presence of these streaks is particularly noticeable in the case of MBP (Fig. 2B, red arrowhead). The signals from both the full-length proteins and the nascent species decreased in intensity after chasing with unlabeled methionine (see Fig. S3A and B), indicating that the interaction of SecA with these newly synthesized polypeptides is transient.

### The interaction of SecA with nascent MBP is not dependent on SecB or TF.

We used the 2-D gel method described above to determine whether SecB or TF was required for the interaction of SecA with nascent Sec substrates. To this end, we examined the interaction of SecA-biotin with nascent polypeptides in strains lacking either SecB (Fig. 2C) or TF (Fig. 2D). The interaction of SecA-biotin was not significantly reduced in either strain, suggesting that neither SecB nor TF is required for the interaction of SecA-biotin with nascent Sec substrates. Indeed, there was a small but reproducible increase in the radioactive signal from both nascent and full-length species in strains lacking SecB (Fig. 2C).

### The interaction of SecB with nascent substrates is dependent on the interaction of SecA with the ribosome.

**FIG 3:**
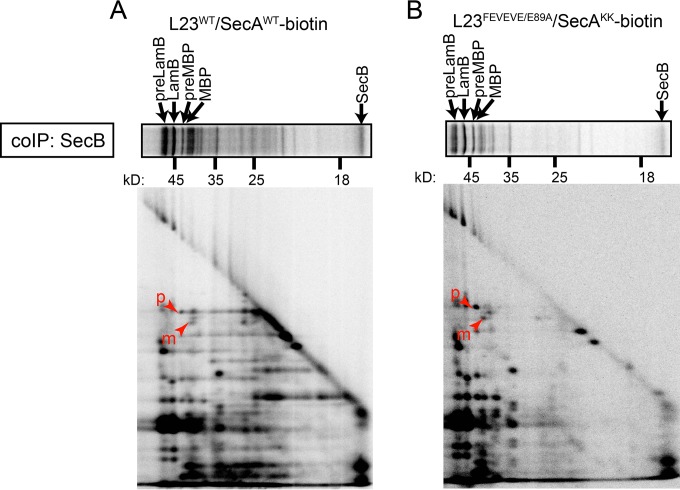
The interaction of SecB with nascent substrates is dependent on the interaction of SecA with the ribosome *in vivo*. (A) Cells expressing wild-type L23 and wild-type SecA-biotin (DRH847) or (B) cells expressing L23^FEVEVE/E89A^ and SecA^KK^-biotin (DRH933) were grown in M63 maltose containing IPTG and pulse-labeled with ^35^S-methionine, and SecB was immunoprecipitated from the cell lysates using specific antiserum. The coprecipitating proteins were separated by size using SDS-PAGE, gel slices of the lanes were subjected to in-gel proteolysis using the V8 protease, and the fragments were resolved in a second dimension in a second round of SDS-PAGE. The first dimension is depicted horizontally above. The running positions of full-length preLamB, mature LamB, preMBP, mature MBP, and SecB in the first dimension are indicated by arrows. The running positions of N-terminal proteolytic fragments of preMBP (p) and mature MBP (m) are indicated by arrowheads in the second dimension.

In order to investigate the dependence of SecB on SecA for interaction with nascent Sec substrates, we carried out complementary 2-D gel experiments in which we immunoprecipitated SecB instead of SecA (Fig. 3A). The pattern of full-length and nascent polypeptides that coimmunoprecipitated with SecB from cells expressing wild-type SecA-biotin was nearly identical to previously published results (23). However, the interaction of SecB with nascent species was greatly reduced in strains in which the interaction between SecA and the ribosome was partially defective (Fig. 3B) (32). This mutant contains alanine substitutions at positions 51, 52, 54, 56, and 89 in ribosomal protein L23 (L23^FEVEVE/E89A^) and at positions 625 and 633 in SecA (SecA^KK^) that reduce but do not eliminate the interaction of SecA with the ribosome (32). These results suggest that the interaction of SecB with nascent substrates is dependent on the interaction of SecA with the ribosomes.

### Interaction of SecA with RNCs *in vitro*.

**FIG 4:**
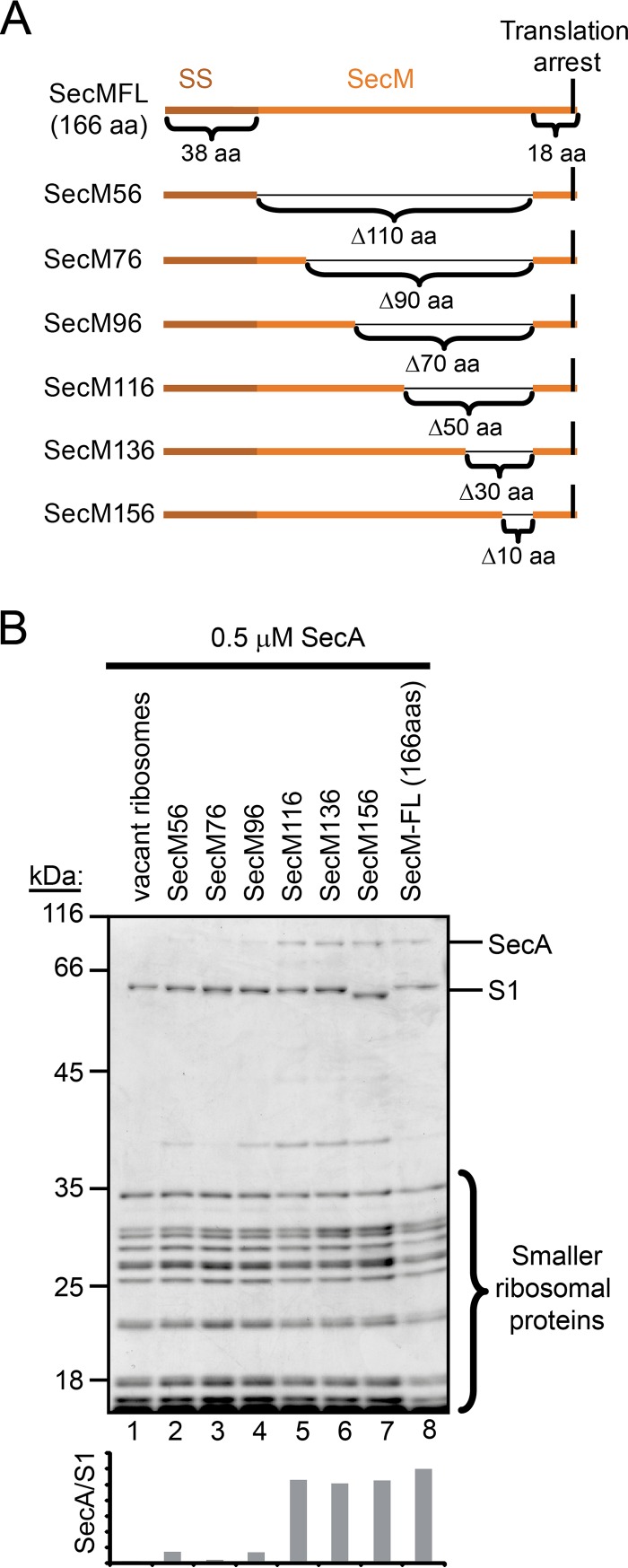
SecA interacts with nascent chains when they reach a length of >116 amino acids. (A) Diagram of the arrested SecM-PhoA fusions. The signal sequence, the 18-amino-acid (aa) amino arrest sequence, and the deleted regions are indicated. Numbering refers to the number of amino acids from the start codon to the arrest-inducing proline in the SecM arrest sequence. (B) SecA (0.5 μM) was incubated with the indicated ribosome-nascent chain complex (RNC; 0.5 μM) in the presence of 250 mM potassium acetate. Ribosomes were subsequently pelleted through a 30% sucrose cushion at >200,000 × *g*, and the pellet fractions were analyzed by SDS-PAGE and Coomassie staining. The running positions of SecA and the ribosomal proteins are indicated. Quantitation of the SecA signal relative to the S1 signal in the Coomassie-stained gel for each RNC is indicated below.

We next examined the interaction of SecA with purified ribosomes containing arrested SecM polypeptides of various lengths *in vitro*. SecM contains an N-terminal signal sequence that targets it for SecA-mediated translocation across the membrane. In addition, it contains a short peptide sequence near the C terminus that stably arrests translation (39, 40). Normally, translocation of SecM across the cytoplasmic membrane overcomes translation arrest and regulates the expression of the downstream *secA* gene (41). The binding of SecA to purified, vacant 70S ribosomes was sensitive to the presence of salt, but the presence of nascent full-length SecM (166 amino acids) stabilized binding in the presence of high salt concentrations, consistent with previous reports (32). In addition, we made a series of internal deletions in the *secM* coding sequence, resulting in different lengths of the N terminus of SecM being fused directly to the C-terminal 18-amino-acid translation arrest sequence (Fig. 4A), in order to examine the ability of SecA to bind to ribosome-nascent chain complexes (RNCs) containing arrested nascent chains with lengths of 56, 76, 96, 116, 136, and 156 amino acids. SecA displayed salt-resistant binding to RNCs when the nascent SecM peptides were 116 amino acids or longer (Fig. 4B). In addition, SecA displayed salt-resistant binding to ribosomes containing MBP, DsbA, and FtsQ nascent chains that were 122 and 182 amino acids but not to ribosomes containing nascent chains that were 62 amino acids long (see Fig. S4A and B, top, in the supplemental material). These results suggest that the minimum length of the nascent chain required for stable interaction with SecA is between 96 and 116 amino acids.

### Effect of TF on binding of SecA to RNCs.

**FIG 5:**
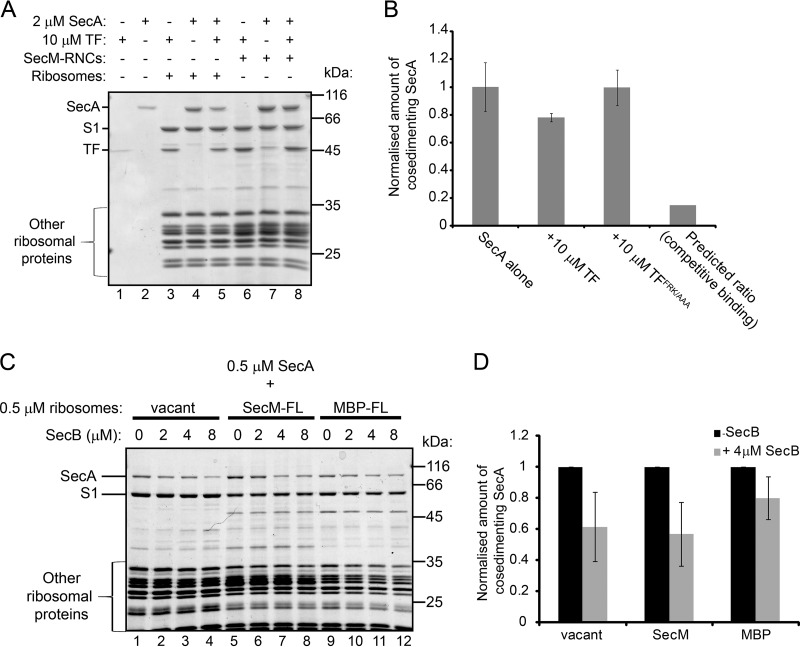
Effects of the presence of TF and SecB on the interaction of ribosome binding by SecA. (A) Vacant 70S ribosomes (1 μM) or SecM-RNCs (1 μM) were incubated with SecA (2 μM) or TF (10 μM) or both, as indicated. After equilibration, binding reaction mixtures were layered on a 30% sucrose cushion and centrifuged at >200,000 × *g*. The ribosomal pellet fractions were resolved by SDS-PAGE and visualized by Coomassie staining. (B) SecA (2 μM) was incubated with vacant 70S ribosomes (1 μM) in the absence or presence of wild-type TF (10 μM) or a ribosome binding-deficient variant, TF^RFK/AAA^ (10 μM). After equilibration, binding reaction mixtures were layered on a 30% sucrose cushion and centrifuged at >200,000 × *g*, and the amount of SecA in the ribosomal pellet relative to the amount of ribosomal protein L1 was determined by quantitative Western blotting. Confidence intervals shown by error bars represent 1 standard deviation from the average of three independent experiments. The predicted ratio is the amount of SecA predicted to cosediment with ribosome in the presence of TF if binding were fully competitive. (C) SecA (0.5 μM) was incubated with vacant 70S ribosomes (0.5 μM), RNCs containing full-length SecM (0.5 μM) or MBP (SecM-FL and MBP-FL, respectively) (0.5 μM), or MBP-FL RNCs (0.5 μM). After equilibration, binding reaction mixtures were layered on a 30% sucrose cushion and centrifuged at >200,000 × *g*. The ribosomal pellet fractions were resolved by SDS-PAGE and visualized by Coomassie staining. (D) Quantitation of the results shown in panel C. The amounts of SecA that cosedimented with the indicated ribosomes in the absence and presence of 4 μM SecB were quantified by densitometry of the Coomassie-stained gel and normalization to the signal from the band corresponding to ribosomal protein S1. Confidence intervals shown by error bars represent one standard deviation from the average of three independent experiments.

TF and SecA interact with different sites on ribosomal protein L23, suggesting that they could compete for binding to nascent polypeptides. The presence of saturating concentrations of TF (present at 10 μM or 20 μM) reduced the amount of SecA (present at 2 μM and 1 μM, respectively) that cosedimented with vacant ribosomes (Fig. 5A, lanes 3 to 5, and B). This reduction was the result of competition between SecA and TF for binding to the ribosome, since a variant of TF containing alanine substitutions at positions 44, 45, and 46 that is defective for binding to the ribosome (TF^FRK/AAA^) did not interfere with the ribosome binding activity of SecA (Fig. 5B) (31). However, the amount of SecA that cosedimented with vacant ribosomes was much higher than expected if binding were mutually exclusive (Fig. 5B) (31, 32). Furthermore, the presence of nascent substrate protein stabilized the binding of both SecA and TF to the ribosome, even in the presence of a large molar excess of TF (Fig. 5A, lanes 6 to 8), indicating that both proteins can bind simultaneously to ribosomes containing nascent substrate proteins.

### Effect of SecB on binding of SecA to RNCs.

The minimum length of a nascent chain required for interaction with SecB is ∼180 amino acids (42). Consistent with these results, SecB displayed an increased affinity for RNCs containing SecM-arrested full-length MBP (396 amino acids in length) compared to its affinity for vacant ribosomes, but the affinity of SecB for nascent MBP that was 122 amino acids in length (MBP122) was comparable to that of vacant ribosomes (see Fig. S4B in the supplemental material). In contrast, SecA displayed increased affinity for RNCs containing both MBP122 and arrested full-length MBP (396 amino acids). The presence of SecB interfered with the ability of SecA to cosediment with both vacant ribosomes and RNCs (Fig. 5C and D; see also Fig. S5). The ability of SecB to interfere with binding of SecA to the ribosomes was independent of its own affinity for the ribosome, suggesting that this activity is the result of the interaction of SecB with SecA.

## DISCUSSION

**FIG 6:**
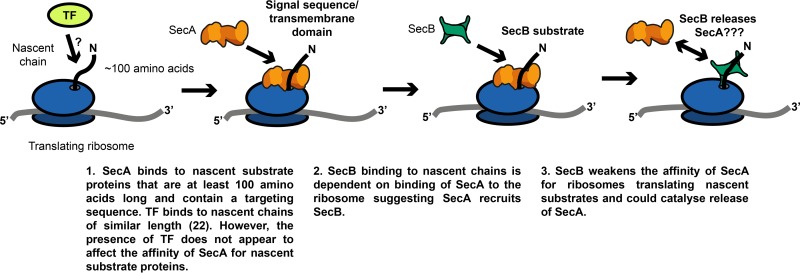
Diagram summarizing the conclusions presented in the Discussion.

Our results indicate that SecA interacts with a broad range of nascent Sec substrate proteins *in vivo* (a diagrammatic summary can be found in Fig. 6). RT-PCR analysis of mRNAs that copurify with SecA indicates that SecA interacts with nascent chains of several known Sec substrates, including Lpp, OmpF, OmpC, TolC, and PhoE. Our 2-D gel analysis indicates that SecA interacts with nascent MBP and several other nascent polypeptides from an early stage in translation. Nascent substrate proteins appear to interact with SecA long before they interact with SecB or productively engage SecYEG. Biochemical experiments indicate that SecA can interact with much shorter nascent chains than can SecB, and the interaction of SecA with MBP in our 2-D gel analysis occurs long before the signal sequence can be removed from MBP (∼35 kDa) (7, 38), which is an indication that the substrate protein is being translocated through SecYEG. The interaction of SecA with the ribosome (32) could facilitate the interaction of SecA with these nascent substrate proteins. These results raise the possibility that a purely posttranslational translocation pathway (i.e., one in which the nascent substrate protein does not contact SecA until after protein synthesis is complete) does not exist in E. coli.

Microarray analysis of the messages that copurified with SecA indicates that SecA interacts with nascent IMPs. This subset of nascent chains shows a significant overlap with those recently identified as interacting with the SRP (20). However, SecA is required for the insertion of a subset of IMPs (12) and for SRP-dependent, cotranslational translocation of a subset of soluble periplasmic proteins (17), suggesting that this result is not an overexpression artifact. In addition, the strong enrichment of mRNAs encoding proteins containing multiple transmembrane domains indicates that SecA has a higher affinity for these proteins. Previous studies suggest that the affinity of SecA for proteins containing signal sequences was only marginally higher than for those lacking signal sequences (43). Nonetheless, our results suggest that this modest increase in affinity is sufficient to promote the specific interaction of SecA with proteins containing internally encoded targeting sequences.

Previous studies have suggested that SecA and TF could compete for interaction with nascent substrates. For example, TF antagonizes SecA-dependent protein translocation *in vivo* (22, 28–30), and it binds to nascent OMPs, such as OmpF, during ongoing translation *in vivo* (22). In addition, nascent polypeptides begin to interact with TF when they reach a length of ∼100 amino acids *in vivo* (22), which is shorter than the minimum length required for stable interaction with SecA. Finally, both SecA and TF bind to L23 (31, 32), and our results indicate that they compete, albeit incompletely, for binding to vacant ribosomes. However, our results also suggest that the presence or absence of TF does strongly influence the interaction of SecA with MBP and other nascent polypeptides *in vivo*. Furthermore, both TF and SecA can bind simultaneously to RNCs containing nascent SecA substrates *in vitro*. These results suggest that TF inhibits SecA-dependent translocation at some step downstream from the interaction of SecA with nascent substrate proteins.

The effect of SecB on the interaction of SecA with nascent substrate proteins is similarly complex. Translocation of MBP becomes fully posttranslational in strains lacking SecB (44), indicating that SecB plays an important role in cotranslational translocation by the uncoupled translocation pathway. SecB interacts with nascent substrate proteins *in vivo* (23) and *in vitro* (42), and SecB is required for the translocation of proteins containing defective signal sequences in many *prlA* suppressor mutants (45–47). In addition, mutations that cause a defect in the interaction between SecA and SecB cause accumulation of substrate-bound SecB (27), and SecB can transfer prebound substrate proteins to SecYEG-bound SecA *in vitro* (26). However, because the interaction of SecA is not dependent on SecB, our results indicate that SecB does not deliver nascent substrate proteins to SecA *in vivo*. Furthermore, mutations that lower the affinity of SecA for the ribosome severely disrupt the ability of SecB to interact with nascent substrate proteins, suggesting that, at least in the case of nascent Sec substrates, SecA targets substrate proteins to SecB. Thus, SecB also appears to function downstream from recognition by SecA in determining the timing of translocation in the uncoupled pathway. One possibility is that SecB is required to release a stable complex formed between SecA and the ribosome when SecA interacts with nascent substrate proteins.

## MATERIALS AND METHODS

### Chemicals and media.

All chemicals were purchased from Roth (Karlsruhe, Germany) or Sigma-Aldrich (St. Louis, MO) except where indicated below. Antiserum against ribosomal protein L23 was a gift from R. Brimacombe, and antiserum against LamB was a kind gift from T. Silhavy. Anti-MBP antiserum was obtained from New England BioLabs (Ipswich, MA). Alkaline phosphatase-coupled anti-rabbit secondary antibody and alkaline phosphatase-coupled StrepTactin were obtained from Vector (Burlingame, CA) and IBA (Göttingen, Germany), respectively. IR700-coupled anti-rabbit and IR800-coupled anti-sheep antibodies were obtained from Rockland (Philadelphia, PA). Cells were grown in lysogeny broth (LB) or M63 minimal medium supplemented with 0.2% maltose (48), as indicated. Where indicated, isopropylthiogalactoside (IPTG; 100 μM) and arabinose (0.1%) were added to the culture medium. Ampicillin (200 μg/ml or 30 μg/ml), kanamycin (30 μg/ml), chloramphenicol (50 μg/ml), and spectinomycin (50 μg/ml) were added where required.

### Strains and plasmids.

Strains and plasmids were constructed using standard methods (48, 49). A list of all the strains and plasmids used in this study can be found in Table S1 in the supplemental material. An amber (TAG) codon was introduced at codon 796 of the *secA* gene in plasmids pDH625 (His-SUMO-SecA) and pDH545 (Strep-SUMO-SecA) using QuikChange (Invitrogen). Variants of *secA* under the control of an IPTG-inducible promoter were introduced onto the chromosome using λInCh (50). pDH733 was constructed by ligating annealed oligonucleotide SecMArrest-for (CATGGGAGACCGGTCCCGGGAGCTCTTCAGCACGCCCGTCTGGATAAGCCAGGCGCAAGGCATCCGTGCTGGCCCTT) and SecMArrest-rev (CCGGAAGGGCCAGCACGGATGCCTTGCGCCTGGCTTATCCAGACGGGCGTGCTGAAGAGCTCCCGGGACCGGTCTCC) and ligating them into plasmid pHK771 (51) cut with NcoI and BspEI. Derivatives of pDH733 were constructed by amplifying the fragment encoding the corresponding portion of SecM or MBP and ligating it into pDH733 cut with NcoI and SacI. Strep-SUMO-SecM-expressing plasmids were constructed by amplifying the SecM-encoding region from the corresponding pDH733 derivative and cloning it into pCA597 cut with BsaI and BamHI.

### Photo-cross-linking of SUMO-SecA-796* and purification of cross-linking products.

The expression of His-SUMO-SecA796* and Strep-SUMO-SecA796* was induced by the addition of 1 mM IPTG, and the proteins were produced in LB medium containing 1 mM Bpa (Bachem) overnight at 18°C. For purification of the His-SUMO-SecA796* cross-linking products, the cells were concentrated and resuspended in 50 mM HEPES potassium salt, pH 7.4, 0.5 mM TCEP [tris(2-carboxyethyl)phosphine], cOmplete EDTA-free protease inhibitor cocktail tablet (Roche). Cross-linking was carried out by exposing the concentrated cells to light at 365 nm on ice. The treated cells were poured into an Avestin-C3 cell disrupter and broken by three passages through the chilled cell. The clarified lysate was supplemented with 500 mM NaCl, 50 mM imidazole and then applied onto a 1-ml HisTrap HP column (GE Healthcare). The column was washed extensively using the same buffer, and the protein was eluted using buffer containing 500 mM imidazole. For Strep-SUMO-SecA796*, cross-linking and lysis were carried out similarly to the procedure for His-SUMO-SecA796* with the exception that 50 mM HEPES potassium salt, pH 7.4, 50 mM potassium acetate, 15 mM magnesium acetate, 1 mM dithiothreitol (DTT) was used as a buffer. The cross-linking products were purified using a 1-ml StrepTrap column (GE Healthcare), and the products were eluted off the column using buffer containing 10 mM desthiobiotin (Sigma-Aldrich).

### LC-MS/MS and N-end sequencing.

Cross-linking products were resolved by SDS-PAGE and excised from a Coomassie-stained gel for liquid chromatography-tandem mass spectrometry (LC-MS/MS) identification (The Advanced Mass Spectrometry Facility, School of Biosciences, University of Birmingham) or N-end sequencing (Alta Biosciences).

### Purification of SecA-cross-linked ribosomes.

Cells producing Strep-SUMO-SecA796* were exposed to UV light at 345 nm and were lysed as described above. Ribosomes were then purified by ultracentrifugation over a 20% sucrose cushion as described previously (31). This served as the total ribosome fraction. Strep-SUMO-SecA796*-cross-linked ribosomes were then purified from the total ribosome fraction using a 1 ml StrepTrap HP column (GE Healthcare) as described above. As a negative control, ribosomes from UV-treated lysates expressing His-SUMO-SecA796* were passed over the column, but no ribosomes could be eluted using 10 mM desthiobiotin, indicating that neither SUMO-SecA nor ribosomes bound nonspecifically to the column.

### RT-PCR.

mRNA was extracted from the total ribosome and Strep-SUMO-SecA796*-cross-linked fractions using an RNeasy minikit (Qiagen). Contaminating DNA was removed from the samples prior to RT-PCR by extensive digestion using a TURBO DNA-free kit (Thermo Fisher). RT-PCR was carried out using the One*Taq* one-step RT-PCR kit (NEB). Reaction mixtures in which the ProtoScript II reverse transcriptase was omitted and replaced with One*Taq* hot start DNA polymerase served as negative controls. Purified E. coli chromosomal DNA was used as a positive control (not shown). The products of the reactions were visualized on Tris-acetate-EDTA-agarose gels.

### Microarray analysis.

Microarray analysis was carried out by the Functional Genomics and Proteomics Service, University of Birmingham, United Kingdom. cDNA was produced by arbitrary PCR using a poly(A) primer from two technical RNA extraction replicates and fluorescently labeled with Cy3. It was then hybridized to an E. coli DNA microarray using the one-color cyanine 3-CTP microarray-based gene expression analysis protocol (Agilent) according to the Agilent low input quick amp labeling kit, version 6. The slide was scanned using an Agilent scanner C and Agilent scan control software version 8.5 at a resolution of 3 μm, and the data were extracted using Agilent Feature Extraction software version 10.10. The processed read value for each E. coli K-12 gene was normalized to the median value for that replicate. The enrichment score was calculated by taking the log_2_ of the signal from the SecA-cross-linked ribosome fraction divided by the signal from the total ribosome fraction. GO categories were based on the annotation in EcoCyc in March 2016. The number of transmembrane domains, largest periplasmic domain, and topology of the N terminus were determined from the annotation for the proteins in UniProtKB. For the analysis, proteins with cytoplasmic N termini were assigned a value of 0, and those with periplasmic N termini were assigned a value of 1. *P* values were calculated using a one-tailed Student's *t* test with a predetermined cutoff for significance of >0.05. The mRNA enrichment data can be found in the Data set S1 in the supplemental material.

### 2-D gel and pulse-labeling experiments.

Cells were grown to an optical density at 600 nm (OD_600_) of 0.5 in M63 minimal medium containing carbon sources as indicated and supplemented with 40 μM biotin and all amino acids except cysteine and methionine. The cultures were then pulse labeled with ^35^S-labeled methionine for 30 s. For examining the temporal processing of MBP and for determining the digestion profile of LamB, MBP or LamB as indicated was immunoprecipitated from the cell lysates using specific antiserum and subjected to 2-D gel analysis as described previously (52). Alternatively, for the analysis of proteins that coprecipitated with SecA or SecB, 2-D gel experiments were carried out similarly to the method of Kumamoto and Francetic (23). Radiolabeling was stopped rapidly by transfer to a ice-water slurry, and cells were lysed by osmotic shock according to the method of Randall and Hardy (53). Cell lysates were incubated with 0.4 mg hydrophilic streptavidin-coupled magnetic beads (New England BioLabs, Ipswich, MA) or with 25 μl protein A-coupled Sepharose beads (GE Healthcare) that had been preincubated with 5 μl rabbit anti-SecB antiserum. In both cases, the bound beads were subsequently washed three times with freshly prepared Tris-buffered saline (TBS) containing 0.1% Tween 20. The purified proteins were eluted by boiling in SDS sample buffer and subjected to 2-D gel analysis as previously described (52).

### Ribosome, RNC, and protein purification.

Ribosomes were purified as described previously (31), and RNCs were purified via an N-terminal tag containing a triple Strep-tag II and SUMO (or via a His_6_ tag in the case of full-length MBP-RNCs) by a method similar to that of Rutkowska et al. (54). SecA, SecB, and trigger factor were purified using an N-terminal His_6_-SUMO tag, as described previously (32). Unless otherwise noted, the SUMO moiety was subsequently cleaved from purified RNCs or proteins by treatment with the SUMO protease, Ulp1, from Saccharomyces cerevisiae.

### Ribosome cosedimentation experiments.

Proteins were incubated with ribosomes or RNCs at the indicated concentrations at 30°C in RNC buffer (10 mM HEPES potassium salt, pH 7.5, 100 mM potassium acetate, 10 mM magnesium acetate, 1 mM β-mercaptoethanol) containing 100 μM ATP. After >10 min, the binding reaction mixture was layered on top of a 30% sucrose cushion made with the same buffer and centrifuged at >200,000 × *g* for 90 min. In order to ensure that the same amounts of ribosomes were loaded in the SDS-PAGE gel, the concentration of ribosomes in the resuspended ribosomal pellet was adjusted according to their absorbance at 260 nm.

### Quantitative Western blotting.

Samples were blotted to nitrocellulose, developed using anti-SecA (rabbit) and anti-L1 (sheep) antibody, and imaged using Li-Cor as described previously (32).

### Software.

Autoradiograms and images of scanned Coomassie-stained gels were analyzed using ImageQuantTL (GE Healthcare), and Western blots were analyzed using the built-in software from Li-Cor (Lincoln, NE).

## Supplementary Material

Supplemental material
