# Supplementary material for: SecA Cotranslationally Interacts with Nascent Substrate Proteins In Vivo
Source: J Bacteriol. 2016 Dec 28;199(2):e00622-16. doi: 10.1128/JB.00622-16 (PMC5198489; doi:10.1128/JB.00622-16)
Supplement: Supplemental material [file supp_199_2_e00622-16__index.html]

SecA Cotranslationally Interacts with Nascent Substrate Proteins In Vivo — Supplemental material 

# SecA Cotranslationally Interacts with Nascent Substrate Proteins *In Vivo*

## Supplemental material

- Supplemental file 1 -

  Fig. S1 (Cross-linking of benzophenone-labeled SecAQ796\* to nascent substrate proteins), S2 (MBP processing and proteolytic profile of LamB), S3 (SecA interaction with nascent substrate proteins), S4 (SecA interaction with nascent chains), and S5 (SecB inhibition of binding of SecA to the ribosome) and Table S1 (Strains and plasmids)

  PDF, 772K
- Supplemental file 2 -

  Data set S1 (Enrichment of mRNAs in SecA-cross-linked ribosomes compared to total ribosomes)

  XLSX, 237K
