# Supplementary material for: SecA Cotranslationally Interacts with Nascent Substrate Proteins In Vivo
Source: J Bacteriol. 2016 Dec 28;199(2):e00622-16. doi: 10.1128/JB.00622-16 (PMC5198489; doi:10.1128/JB.00622-16)
Supplement: Supplemental material [file JB.00622-16_zjb999094280s1.pdf]

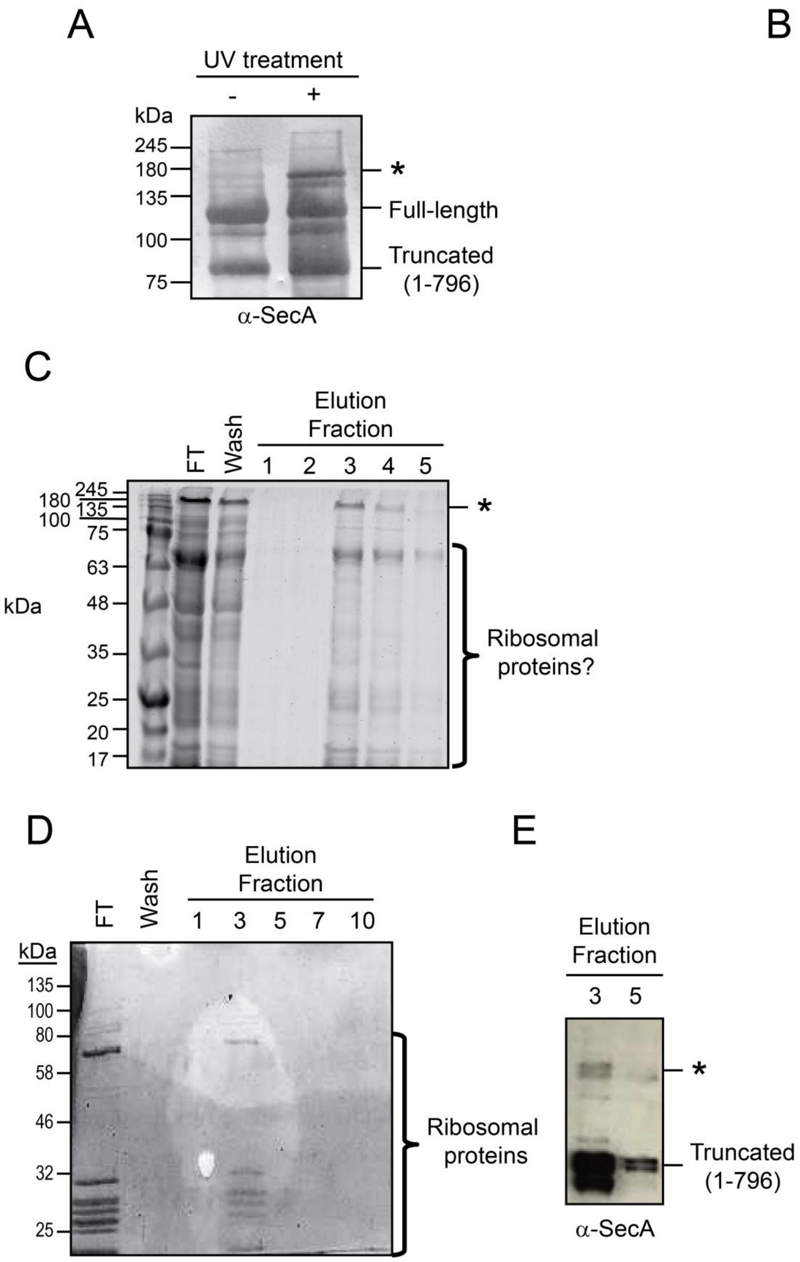

B

Protein	Unique Peptides	MW
SecA	63	102
OmpF	15	39.3
RpoB	43	150.5
ArnA	24	74.2
LamB	13	49.9
TolC	11	53.7
YfhM	14	181.5
RpoC	15	155.1
AtpA	15	55.2
TamB	14	136.7
NuoC	13	68.2
DeaD	9	70.5
GltB	8	163.2
SpoT	4	79.3
OmpC	5	40.3
Ppk	3	80.4
Dld	4	64.6
ZntA	2	76.8
Lpp	2	8.3
MppA	1	59.9
RplO	1	15
SecY	1	48.5
MdoH	1	96.9
YrhB	1	10.6
CyoA	1	34.9
DnaK	1	69.1
Rne	1	118.1
RplQ	2	14.4
RplT	1	13.5
BorD	1	10.4
GspI	1	13.9
MscS	1	30.9
PhoE	1	38.9
SoxS	1	12.9

Figure S1. Benzophenone-labelled SecAQ796* crosslinks to nascent substrate proteins. (A) Anti-SecA western blot of purified photocrosslinked His-SUMO-SecAQ796* from figure 1A in the main text. The running position of the truncated SecA without incorporated Bpa, full-length SecA Bpa and photocrosslinking adducts are indicated. (B) List of proteins identified by LC-MS/MS, which copurify with His-SUMO-SecAQ796* after photocrosslinking. Cell envelope proteins (i.e. IMPs, OMPs, soluble periplasmic proteins and lipoproteins) are highlighted yellow.

The number of unique peptides identified by LC-MS/MS and the molecular weight of each protein are indicated. (C) Cells expressing Strep-SUMO-SecAQ796* were photocrosslinked as described in the text, and lysates were passed over a StretTactin column. Strep-SUMO-SecAQ796* was eluted from the column using buffer containing 10 mM desthiobiotin. The flow through from the cell lysate flow through (FT), the first wash fraction and the elution fractions were resolved on a 15% SDS-PAGE and Coomassie stained. The running positions of the crosslinking adduct (*) and of potential ribosomal proteins are indicated. (D & E) Strep-SUMO-SecAQ796* was photocrosslinked *in vivo* as in (C). Ribosomes were then purified from the cell lysates by ultracentrifugation, and the purified ribosomes were passed over a StrepTactin column. Purified Strep-SUMO-SecAQ796* crosslinked ribosomes were eluted using 10 mM desthiobiotin. The ribosome flow-through (FT), wash and elution fractions were resolved on a 12% SDS-PAGE gel. The gels were Coomassie stained (D) or transferred to a nitrocellulose membrane for western blotting against SecA (E). The running positions of the crosslinking adduct (*) and ribosomal proteins are indicated.

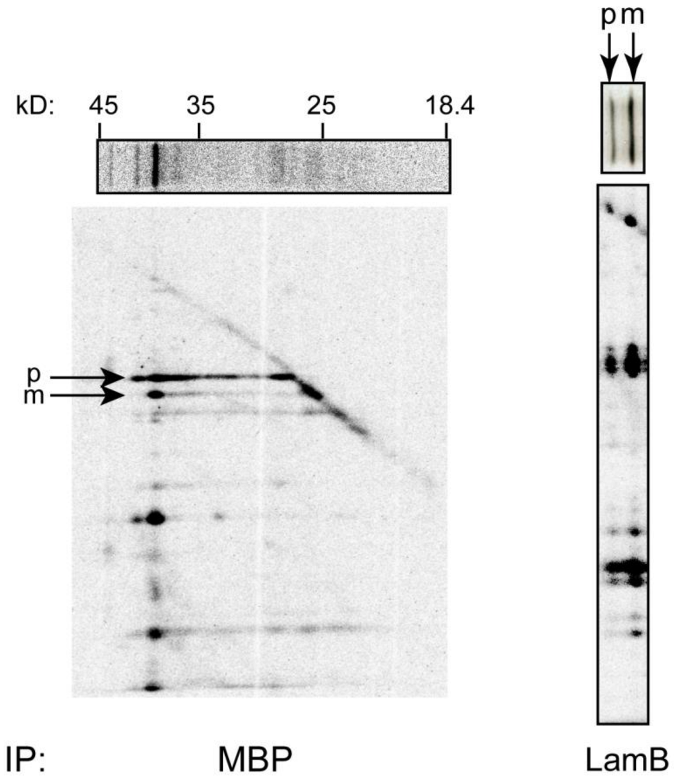

Figure S2. 2D gels of MBP processing and proteolytic profile of LamB. DRH839 were grown in M63 maltose containing IPTG and pulse-labelled with ^{35}S-methionine. MBP or LamB, as indicated, were immunoprecipitated from the cell lysates using specific antiserum and was separated according to size using SDS-PAGE (upper portions of each subfigure). Gel slices of the lane were subjected to in-gel proteolysis using V8 protease and resolved in a second dimension by SDS-PAGE. The running positions of the N-terminal proteolytic fragments of precursor MBP (p) and mature MBP (m) and of full-length precursor LamB (p) and mature LamB (m) are indicated.

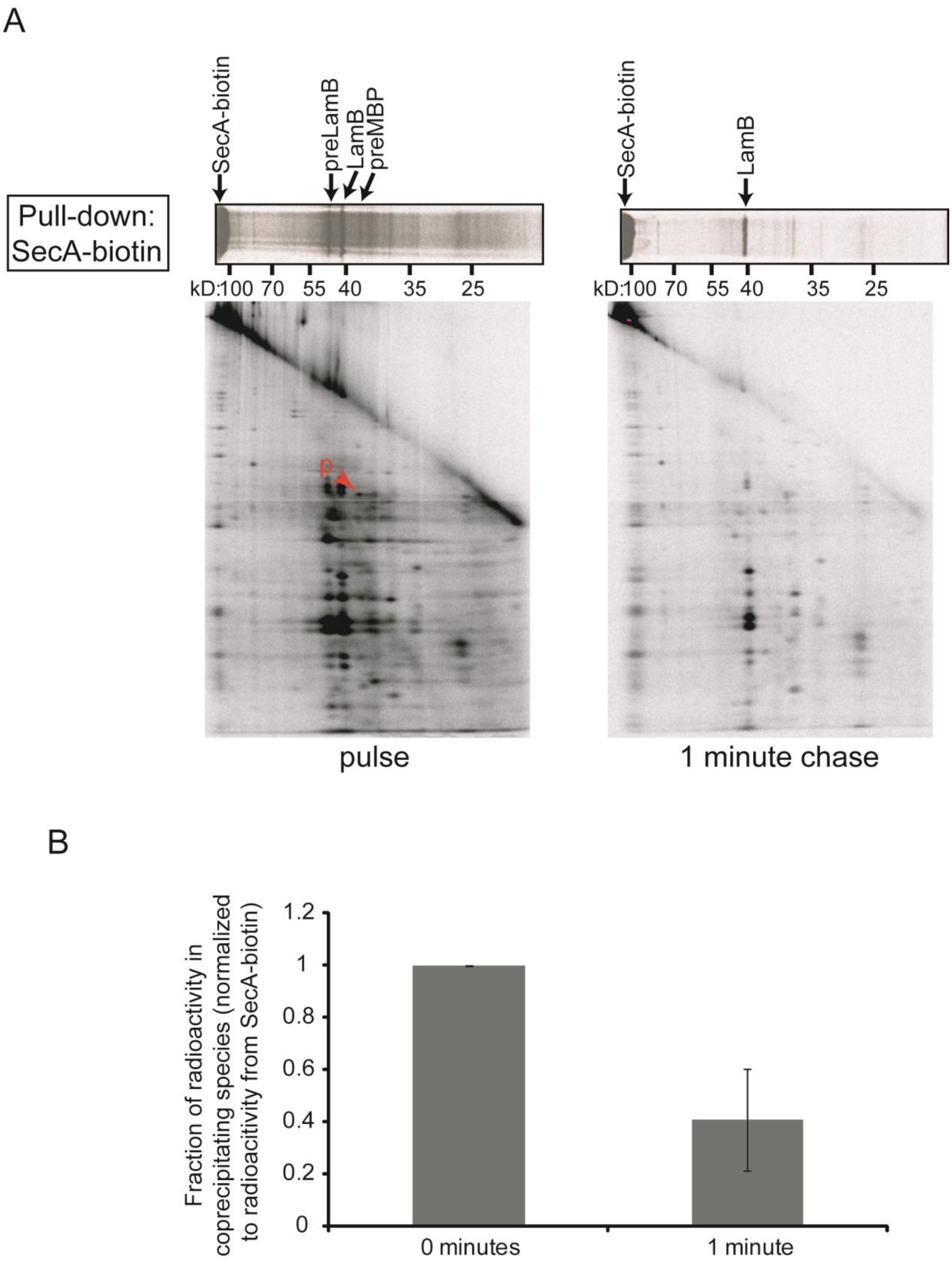
A
Pull-down: SecA-biotin
SecA-biotin
preLamB
LamB
preMBP
kD:100
70
55
40
35
25
SecA-biotin
LamB
kD:100
70
55
40
35
25
p
pulse
1 minute chase
B
Fraction of radioactivity in coprecipitating species (normalized to radioactivity from SecA-biotin)
1.2
1
0.8
0.6
0.4
0.2
0
0 minutes
1 minute

Figure S3. The interaction of SecA with nascent substrate proteins in transient. (A) Strain DRH839 (WT) was grown in M63 maltose and pulse-labeled with ^{35}S-methionine for 30 seconds, and SecA-biotin was pulled down from the cell lysate either immediately (left) or after a 1 minute chase with unlabeled methionine (right). The coprecipitating proteins were resolved using SDS-PAGE as in figure 1 in the main text with the exception that this first dimension was run on a 12% SDS-PAGE gel instead of a 15% gel. Gel slices corresponding to the lane were subjected to in-gel proteolysis using the V8 protease and resolved in a second dimension by SDS-PAGE. The running positions of full length precursor LamB (preLamB), mature LamB (LamB) and precursor MBP (preMBP) in the first dimension are indicated with black arrows. The N-terminal proteolytic fragment of precursor-length MBP (p) is indicated by a red arrowhead. (B) Quantification of the total radioactivity of the immunoprecipitating species normalized to the radioactive signal from SecA-biotin. Confidence intervals indicate the range of values determined.

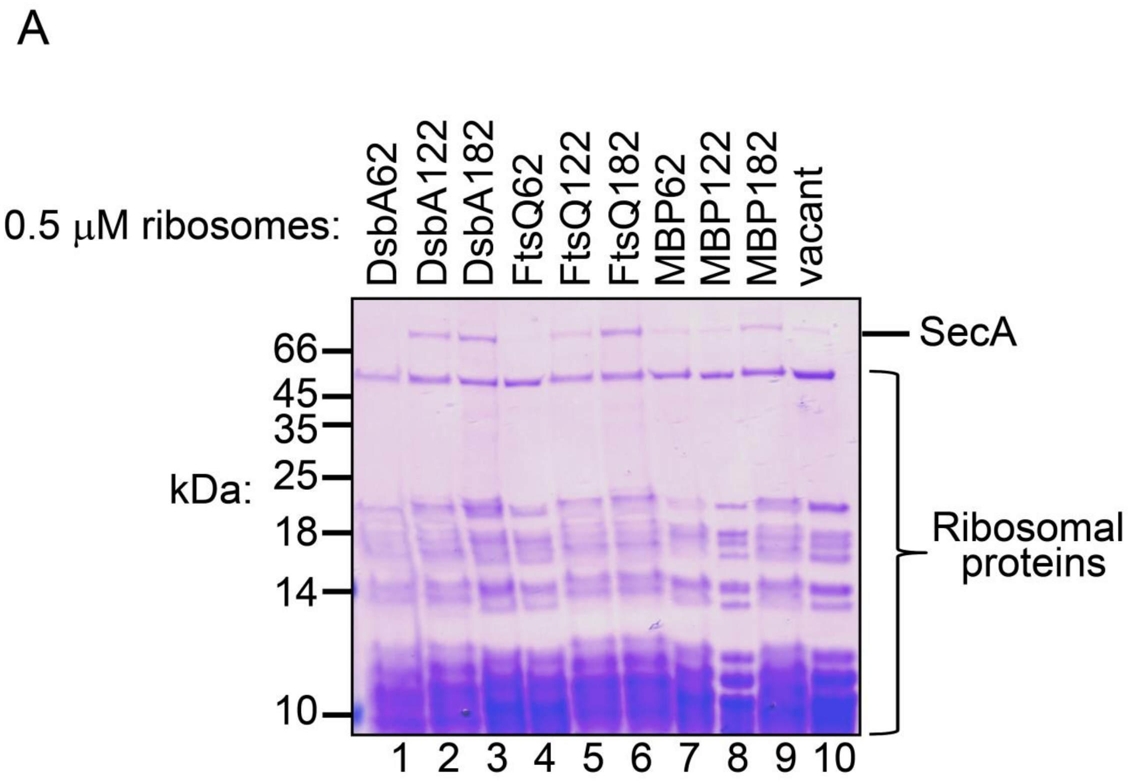

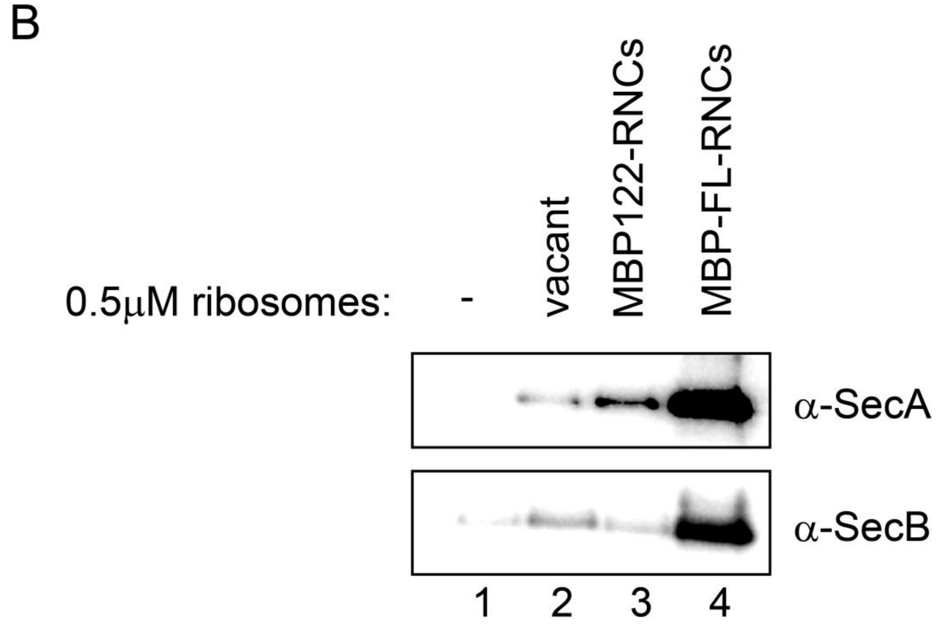

Figure S4. SecA can interact with nascent chains greater than 96 amino acids in length. (A) 0.5μM SecA was incubated with 0.5μM of the indicated ribosome-nascent chain complex (RNC) in the presence of 300mM potassium acetate. Ribosomes were then pelleted through a 30%

sucrose cushion using ultracentrifugation, and the pellet fractions were analyzed by SDS-PAGE and Coomassie staining. Nascent chain lengths of 62 (lanes 1, 4 & 7), 122 (lanes 2, 5 & 8), and 184 (lanes 3, 6 & 9) correspond to 44, 104, and 164 of the N-terminal residues of the indicated protein fused to an 18 amino acid SecM-arrest peptide. The running positions of SecA and the ribosomal proteins are indicated. (B) 0.5µM SecA (above) or SecB (below) was incubated with no ribosomes (lane 1) or 0.5µM vacant ribosomes (lane 2), MBP122 RNCs (lane 3), or MBP-FL (lane 4) RNCs in the presence of 300mM potassium acetate. Ribosomes were then pelleted through a 30% sucrose cushion using ultracentrifugation, and the pellet fractions were analyzed by SDS-PAGE and western blotting against SecA or SecB, as indicated.

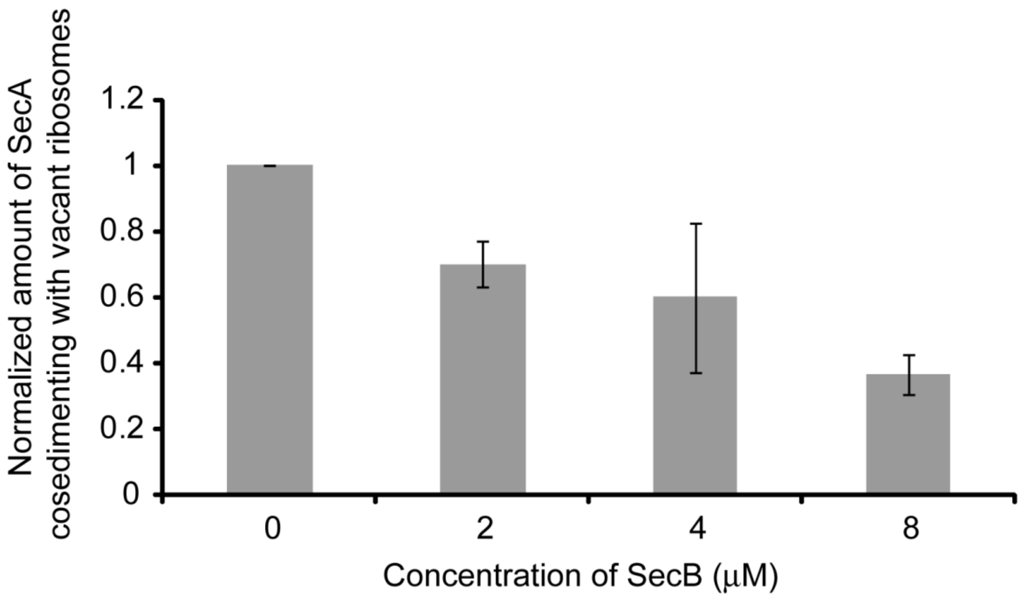

Figure S5. SecB inhibits binding of SecA to the ribosome. Quantification of the amount of SecA cosedimenting with ribosomes in the presence of increasing concentrations of SecB. The amount of SecA the cosedimented with ribosomes was determined by densitometry of Coomassie stained gels and normalised to the signal from ribosomal protein S1. Concentrations of SecB are given for the tetramer. Confidence intervals are the range of values measured for independent experiments.

Table S1. Strains and plasmids.

**Strain**	**Genotype**	**Source**
BL21 DE3	E. coli B F- *ompT gal dcm hsdSB*(*rB- mB-*) λDE3	Laboratory stock
MC4100	F- *araD139* Δ(*argF-lac*)*U169 rpsL150 relA1 deoC1 rbsR fthD5301 fruA25* λ-	Laboratory stock
DRH741	MC4100 Δ*secA* λ-p_{Trc}-SecA(WT) $Spec^R$	This study
DRH744	MC4100 Δ*secA* λ-p_{Trc}-SecA(K625A/K633A) $Spec^R$	This study
DRH839	MC4100 Δ*secA* λ-p_{Trc}-SecA-biotin $Spec^R$	This study
DRH841	DRH839 Δ*secB*::Kan^R	This study
DRH847	DRH839 Δ*rplW*::KanR + pBB6623	This study
DRH866	DRH839 Δ*tig*::Kan^R	This study
DRH889	MC4100 Δ*secA* λ-p_{Trc}-SecA(K625A/K633A)-biotin $Spec^R$	This study
DRH933	DRH889 Δ*rplW*::KanR + pDH630	This study
DRH692	MC4100 Δ*secA*::KanR + pDH692	(1)
DRH940	MC4100 Δ*secA*::KanR + pDH939	This study
**Plasmid**	**Description**	**Source**
pTrc99a/b	multi-copy plasmid containing an IPTG-inducible promoter	Promega
pDSW204	pTrc99a, attenuated promoter, ampicillin resistant	(2)
pBB6623	pTrc99b + *rplW*	(3)
pCA528	pET24a + His_6 + *SMT3* (*Saccharomyces cerevisiae*)	(4)
pCA597	pET24a + StrepTag $(II)_3$ + *SMT3* (*Saccharomyces cerevisiae*)	(4)
pHK771	pTrc99a + PhoA	(5)
pASSS1	pCA597 + SecM	Laboratory stock
pASSS-MalE26	pASSS1 + region encoding N-terminal 26 amino acids of MalE	This study

pASSS-MalE86	pASSS1 + region encoding N-terminal 86 amino acids of MalE	This study
pASSS-MalE146	pASSS1 + region encoding N-terminal 146 amino acids of MalE	This study
pASSS-DsbA26	pASSS1 + region encoding N-terminal 26 amino acids of DsbA	This study
pASSS-DsbA86	pASSS1 + region encoding N-terminal 86 amino acids of DsbA	This study
pASSS-DsbA146	pASSS1 + region encoding N-terminal 146 amino acids of DsbA	This study
pASSS-FtsQ26	pASSS1 + region encoding N-terminal 26 amino acids of FtsQ	This study
pASSS-FtsQ86	pASSS1 + region encoding N-terminal 86 amino acids of FtsQ	This study
pASSS-FtsQ146	pASSS1 + region encoding N-terminal 146 amino acids of FtsQ	This study
pDH585	pCA528 + SecB	This study
pDH625	pCA528 + SecA(WT)	(1)
pDH630	pTrc99b + *rplW*(FEVEVE→AAVAVA/E89A)	(1)
pDH692	pDSW204 + SecA	(1)
pDH784	pCA597 + $SecM^{1-38}$-$SecM^{148-166}$	This study
pDH785	pCA597 + $SecM^{1-58}$-$SecM^{148-166}$	This study
pDH786	pCA597 + $SecM^{1-78}$-$SecM^{148-166}$	This study
pDH787	pCA597 + SecM	(1)
pDH788	pCA597 + $SecM^{1-98}$-$SecM^{148-166}$	This study
pDH789	pCA597 + $SecM^{1-118}$-$SecM^{148-166}$	This study
pDH790	pCA597 + $SecM^{1-138}$-$SecM^{148-166}$	This study
pDH894	pCA528 + *malE* (full length) + $SecM^{148-166}$	This study
pMJ101	pCA528 + *secA*(Q975Amber)	This study
pMJ102	pCA597 + *secA*(Q975Amber)	This study

References

1. **Huber D, Rajagopalan N, Preissler S, Rocco MA, Merz F, Kramer G, Bukau B.** 2011. SecA interacts with ribosomes in order to facilitate posttranslational translocation in bacteria. Mol Cell **41:**343-353.
2. **Weiss DS, Chen JC, Ghigo JM, Boyd D, Beckwith J.** 1999. Localization of FtsI (PBP3) to the septal ring requires its membrane anchor, the Z ring, FtsA, FtsQ, and FtsL. J Bacteriol **181:**508-520.
3. **Kramer G, Rauch T, Rist W, Vorderwulbecke S, Patzelt H, Schulze-Specking A, Ban N, Deuerling E, Bukau B.** 2002. L23 protein functions as a chaperone docking site on the ribosome. Nature **419:**171-174.
4. **Andreasson C, Fiaux J, Rampelt H, Mayer MP, Bukau B.** 2008. Hsp110 is a nucleotide-activated exchange factor for Hsp70. J Biol Chem **283:**8877-8884.
5. **Kadokura H, Beckwith J.** 2009. Detecting folding intermediates of a protein as it passes through the bacterial translocation channel. Cell **138:**1164-1173.
